# Tomato hypersensitivity in peach allergic patients: rPru p3 and rPru p1 positivity is predictive of the symptom severity

**DOI:** 10.1186/2045-7022-1-S1-P77

**Published:** 2011-08-12

**Authors:** Marta Piantanida, Larua Farioli, Joseph Scibilia, Ambra Mascheri, Valerio Pravettoni, Laura Primavesi, Michele Nichelatti, Alessandro Marocchi, Jan Walter Schroeder, Chrysi Stafylaraki, Elide Anna Pastorello

**Affiliations:** 1 Foundation IRCCS Ca’ Granda Ospedale Maggiore Policlinico, Clinical Allergy and Immunology Unit, Milan, Italy; 2 Niguarda Hospital, Laboratory Medicine, Milan, Italy; 3 Niguarda Hospital, Allergology and Immunology, Milan, Italy; 4 Niguarda Hospital, Center for Clinical Research, Milan, Italy

## Background

allergy to tomato is very common in the Mediterranean area, where tomato consumption has increased in the last years: fresh in salads, cooked in household sauces or industrially processed foods. Many patients allergic to tomato present severe reactions and have symptoms also with commercial tomato products.

## Objective

to examine the possibility of a relationship between severe allergic symptoms to tomatoes and peach; and, in the event of such a relationship, examine the correlation between severe tomato allergy and IgE positivity to peach allergens, i.e. Pru p 3 and Pru p 1.

## Methods

within a population of patients with different grades of OAS for peach (mild-OAS, group A; and severe systemic symptoms to peach, group B) we selected patients with documented allergic reactions to tomato. We investigated the type of reaction to tomato by means of a clinical questionnaire, skin prick tests, prick + prick with fresh tomato and anti-rPru p 1, 3, 4, anti-rBet v 1, 2 and 4 IgE specific measurements. We compared the kind of clinical reactions to tomato between group A and group B patients. Statistical analyses were carried out with parametric and non parametric tests to examine the relationship between anti-rPru p 1 and 3 IgE levels and symptom severity.

## Results

patients with severe symptoms to peach (group B) were at major risk of presenting severe symptoms to tomato (p=0.017). We investigated this correlation and found that patients with systemic severe symptoms to tomato presented higher specific IgE levels to rPru p 3 than patients with mild OAS (p= 0.0291). On the contrary patients with mild OAS to tomato presented higher rPru p 1 specific IgE levels than patients with severe systemic symptoms (p=0.047).

## Conclusions

peach and tomato allergy are strictly interrelated and IgE positivity to rPru p 3 can be considered a biological marker of severe symptoms to tomato, whereas IgE to rPru p1 can be considered a marker for milder symptoms.

